# Publisher Correction: Essential roles of plexin-B3^+^ oligodendrocyte precursor cells in the pathogenesis of Alzheimer’s disease

**DOI:** 10.1038/s42003-021-02905-5

**Published:** 2021-12-02

**Authors:** Naomi Nihonmatsu-Kikuchi, Xiu-Jun Yu, Yoshiki Matsuda, Nobuyuki Ozawa, Taeko Ito, Kazuhito Satou, Tadashi Kaname, Yasushi Iwasaki, Akio Akagi, Mari Yoshida, Shuta Toru, Katsuiku Hirokawa, Akihiko Takashima, Masato Hasegawa, Toshiki Uchihara, Yoshitaka Tatebayashi

**Affiliations:** 1 Affective Disorders Research Project, Tokyo Metropolitan Institute of Medical Science, Setagaya, Tokyo Japan; 2 Department of Neurology, Key Laboratory of Neurology of Hebei Province, Second Hospital of Hebei Medical University, Shijiazhuang, China; 3 Department of Genome Medicine, National Center for Child Health and Development, Setagaya, Tokyo Japan; 4 Institute for Medical Science for Aging, Aichi Medical University, Nagakute, Aichi Japan; 5 Department of Neurology, Nitobe Memorial Nakano General Hospital, Nakano, Tokyo Japan; 6 Department of Life Science, Gakushuin University Graduate School of Science, Toshima, Tokyo Japan; 7 Dementia Research Project, Tokyo Metropolitan Institute of Medical Science, Setagaya, Tokyo Japan

**Keywords:** Mechanisms of disease, Alzheimer's disease, Brain injuries, Neurodegeneration, Alzheimer's disease

Correction to: *Communications Biology* 10.1038/s42003-021-02404-7, published online 15 July 2021.

Figure 4 was missing from the PDF of this article. The original PDF has been corrected. The HTML version of the Article was correct at the time of publication.

