# Supplementary material for: Essential roles of plexin-B3+ oligodendrocyte precursor cells in the pathogenesis of Alzheimer’s disease
Source: Commun Biol. 2021 Jul 15;4:870. doi: 10.1038/s42003-021-02404-7 (PMC8282672; doi:10.1038/s42003-021-02404-7)
Supplement: Supplementary file 2 — SUPPLEMENTAL MATERIAL [file 42003_2021_2404_MOESM2_ESM.pdf]

Supplementary materials

Essential roles of plexin-B3$^+$ oligodendrocyte precursor cells in the pathogenesis of Alzheimer's disease

Naomi Nihonmatsu-Kikuchi, Xiu-Jun Yu, Yoshiki Matsuda, Nobuyuki Ozawa, Taeko Ito, Kazuhito Satou, Tadashi Kaname, Yasushi Iwasaki, Akio Akagi, Mari Yoshida, Shuta Toru, Katsuiku Hirokawa, Akihiko Takashima, Masato Hasegawa, Toshiki Uchihara, Yoshitaka Tatebayashi

Contents:

Supplementary Figures 1 - 16

Supplementary Tables 1 - 4

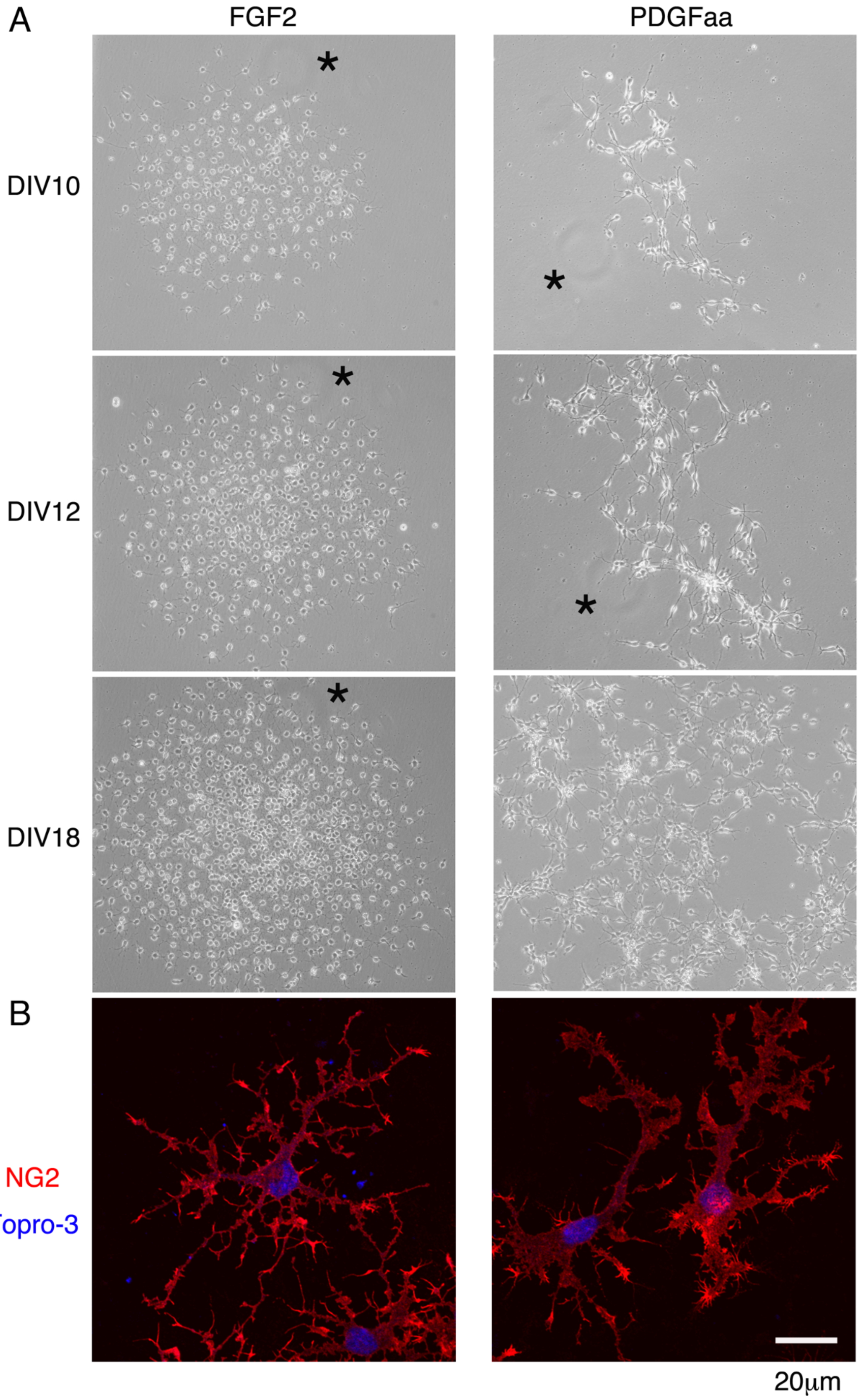
A
FGF2
PDGFaa
DIV10
DIV12
DIV18
B
NG2
Topro-3
20μm

Supplementary Figure 1. Effects of FGF2 or PDGF-aa on NG2^{+} aOPCs in the primary cultures.

(**A**) Phase contrast images of primary cultures in medium containing FGF2 (20 ng/ml) or PDGF-aa (20 ng/ml). Phase contrast images at 10, 12, and 18 days in vitro (DIV) show typical colony formations. While cell density was higher in FGF2 and the cells migrated slowly, the cells were relatively scattered and migrated faster in PDGF-aa. Note that in passaged cultures, PDGFaa no longer renders NG2^{+} aOPCs proliferative (see **Figure 1d**). Asterisks indicate position markers. Scale bar: 300 μm.

(**B**) Morphology of NG2^{+} cells in medium containing FGF2 (20 ng/ml; left panel) or PDGF-aa (20 ng/ml; right panel). The cells were stained using an anti-NG2 antibody (red) and TO-PRO-3 (blue). The typical morphologies of NG2^{+} OPCs in FGF2 were multipolar, while those in PDGF-aa were generally bipolar. Scale bar: 20 μm.

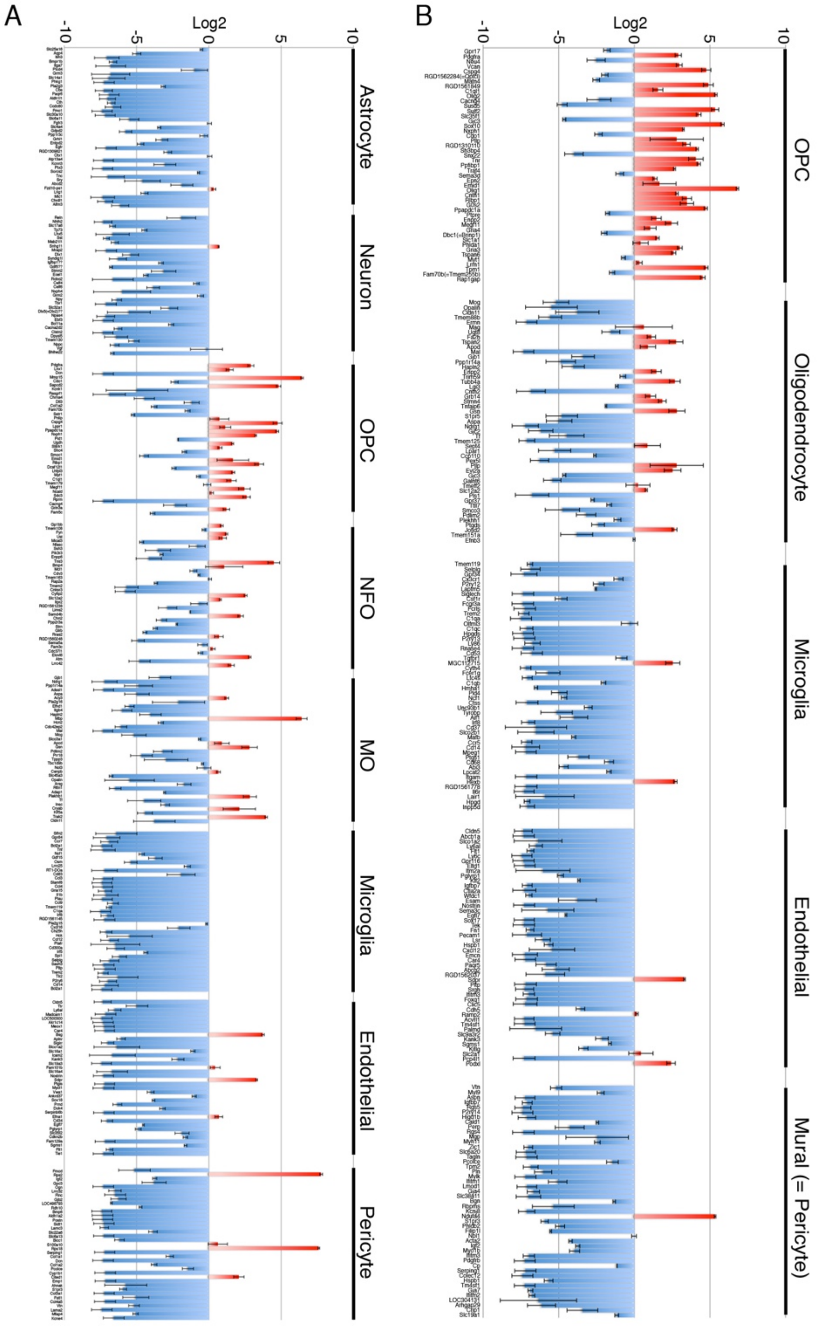

Supplementary Figure 2. Microarray profiles of cultured aOPCs (n = 3).

(**A**) Comparison with the top 40 cell type-specific genes reported in the study by Zhang et al.[25] Data for the top 40 astrocyte, neuron, OPC, newly formed oligodendrocyte (NFO), myelinating oligodendrocyte (MO), microglia, endothelial cell, and pericyte genes from the adult mouse cortex[25] were compared with the microarray data obtained from cultured aOPCs. Note that cultured aOPCs generally express oligodendrocyte lineage (OPC, NFO, and MO) specific genes, especially those specific to OPCs.

Although the reason is unclear, two pericyte specific genes, ribosomal protein S2 (Rps2) and Rps18, were highly enriched in cultured aOPCs. For the complete list of the genes, also see **Supplementary Data 2**. Red bars: highly expressed genes; blue bars: downregulated genes.

(**B**) Comparison with the top 50 genes reported in the study by Wu et al.[26] Data for the top 50 OPC, oligodendrocyte, microglia, endothelial cell, and mural cell (pericyte) genes obtained from single-cell RNA-seq of 8–10-month old mouse amygdala[26] were compared with the microarray data obtained from cultured aOPCs. See also **Supplementary Data 3**. Red bars: highly expressed genes; blue bars: downregulated genes.

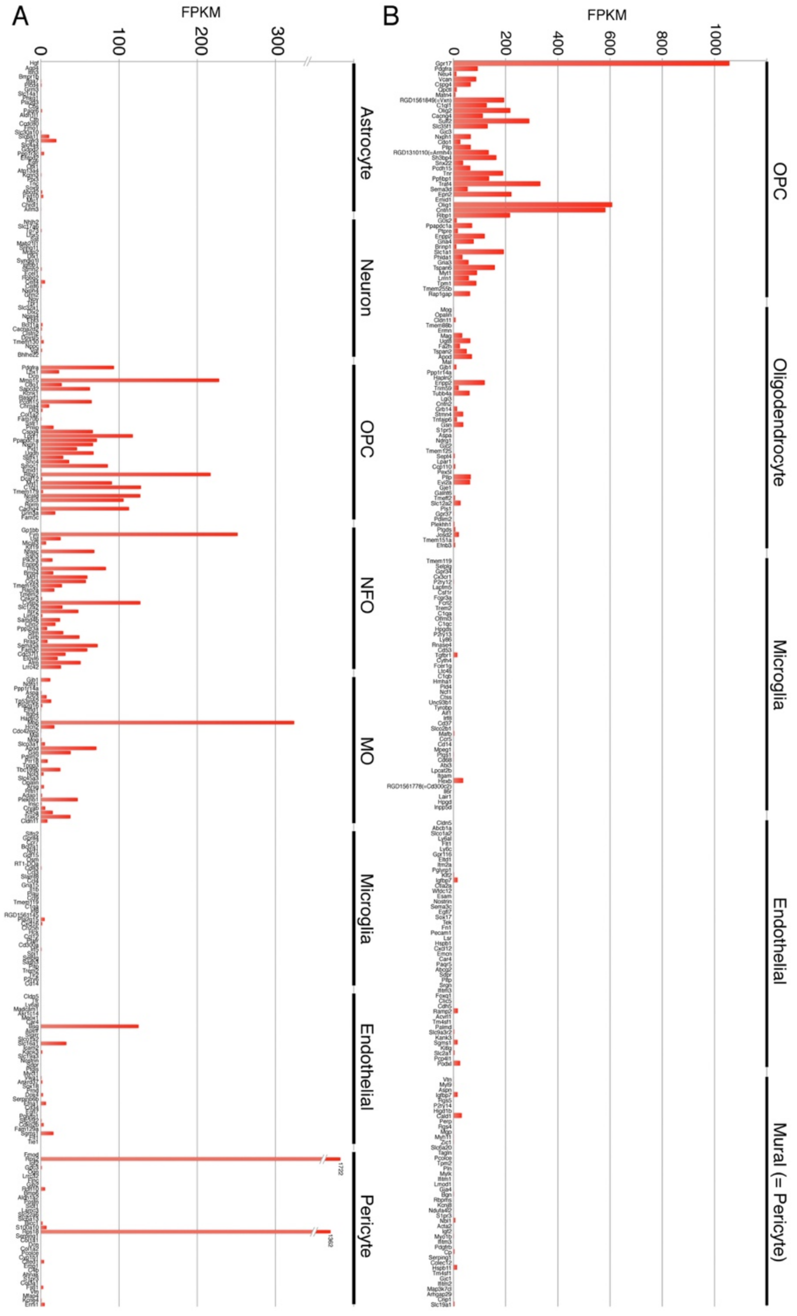

Supplementary Figure 3. RNA-seq profiles of cultured aOPCs (n = 2).
(**A**) Comparison with the top 40 cell type-specific genes reported by Zhang et al.[25] Data for the top 40 astrocyte, neuron, OPC, NFO, myelinating oligodendrocyte, microglia, endothelial cell, and pericyte genes from the adult mouse cortex[25] were compared with the RNA-seq data obtained from cultured aOPCs. Note that cultured aOPCs express OPC specific-genes. As observed in the microarray analysis, two pericyte specific genes, Rps2 and Rps18, were highly enriched in cultured aOPCs. See also **Supplementary Data 5**.

(**B**) Comparison with the top 50 genes reported by Wu et al.[26] Data for the top 50 OPC, oligodendrocyte, microglia, endothelial cell, and mural cell (pericyte) genes obtained from single-cell RNA-seq of 8–10-month old mouse amygdala[26] were compared with the RNA-seq data obtained from cultured aOPCs. Note that cultured aOPCs express OPC specific-genes. See also **Supplementary Data 6**.

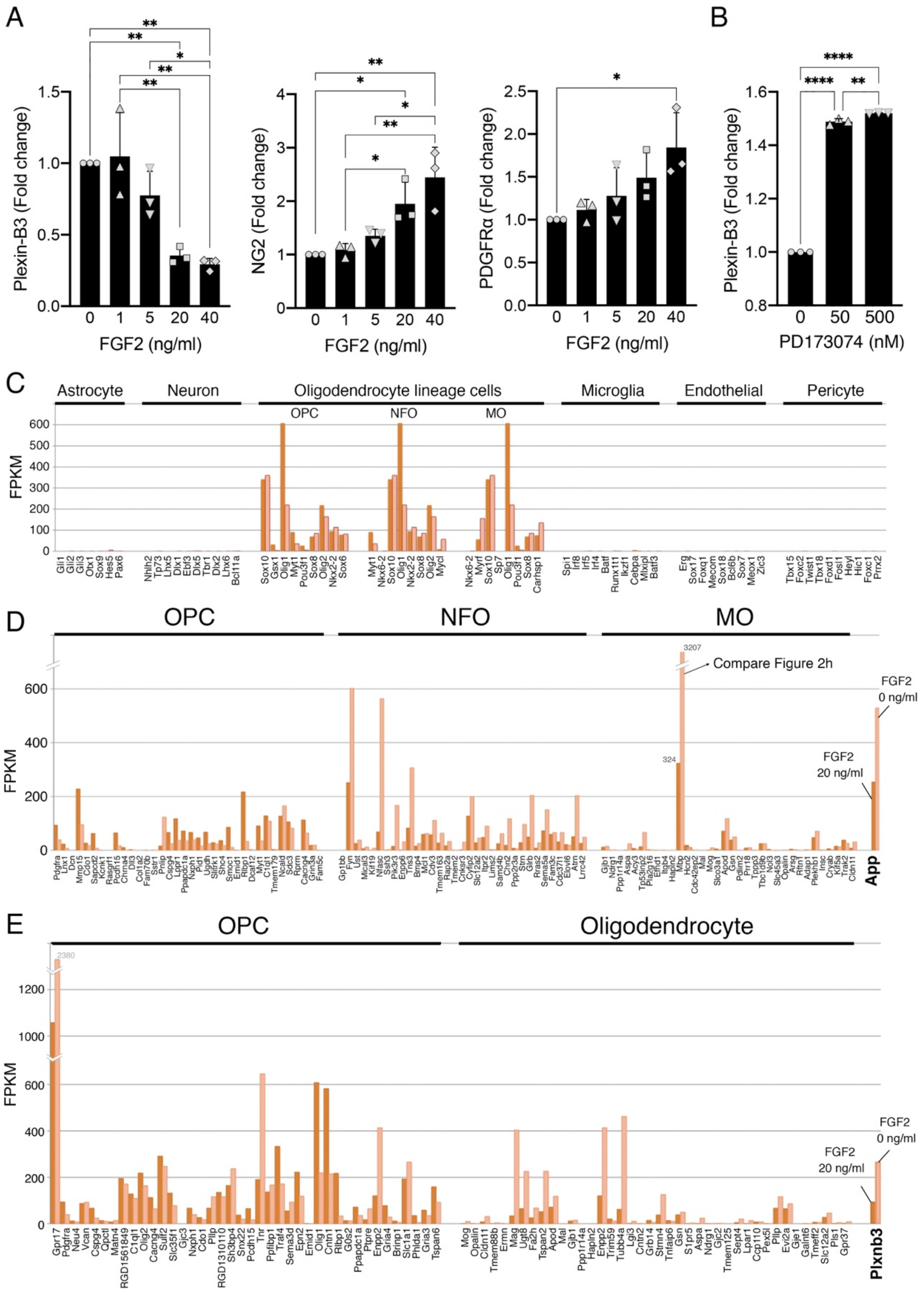

Supplementary Figure 4. Effects of FGF2 withdrawal on cultured aOPCs.

(**A**) Quantification of the levels of plexin-B3, NG2, and PDGFRα in **Figure 2d** (n = 3/group; $^{**}P < 0.01$, $^{*}P < 0.05$; one-way ANOVA, Tukey's multiple comparisons test. The protein levels are calculated as those at 0 ng/ml FGF2 as 1).
(**B**) Quantification of the levels of plexin-B3 in **Figure 2f**. To analyze the effects of FGF2, the cells were cultured in medium containing FGF2 inhibitor PD173074 (0, 50, or 500 nM in the presence of 20 ng/ml FGF2; Merck, Darmstadt, Germany) for 24 h. Note that PD173074 dose-dependently increased plexin-B3 levels in cultured aOPCs (n = 3/group; $^{****}P < 0.0001$, $^{**}P < 0.01$; one-way ANOVA, Tukey's multiple comparisons test. The protein levels are calculated as those at 0 nM PD173074 as 1).
(**C**) Effects of FGF2 withdrawal on the transcription factor gene expression profiles of cultured aOPCs. Data for the top 10 astrocyte, neuron, OPC, NFO, myelinating oligodendrocyte, microglia, endothelial cell, and pericyte transcription factor genes reported by Zhang et al.[25] were compared with the RNA-seq data obtained from aOPCs cultured in medium with or without 20 ng/ml FGF2 (n = 2). Note that the 5-day FGF2 withdrawal did not change the transcription factor gene expression. For the complete list of the genes, see **Supplementary Data 4**.
(**D**) Effect of 5-day FGF2 withdrawal on the RNA-seq profiles of cultured aOPCs. Data for the top 40 OPC, NFO, and MO genes reported by Zhang et al.[25] were compared with the RNA-seq data of aOPCs cultured with or without 20 ng/ml FGF2 (n = 2). Note that 5-day FGF2 withdrawal increased the RNA levels for several NFO-specific genes and one MO-specific gene (MBP) in cultured aOPCs (compare MBP protein levels in **Figure 2h**). For the complete list of the genes, see **Supplementary Data 5**. The right-most bars show the change in App gene expression.
(**E**) Effect of 5-day FGF2 withdrawal on the RNA-seq profiles of cultured aOPCs. Data for the top 40 OPC and oligodendrocyte genes reported by Wu et al.[26] were compared with the RNA-seq data of aOPCs cultured with or without 20 ng/ml FGF2 (n = 2). Note that 5-day FGF2 withdrawal increased or decreased the RNA levels of several OPC- and oligodendrocyte-specific genes in cultured aOPCs. See also **Supplementary Data 6**. The right-most bars show the change in Plxnb3 gene expression.

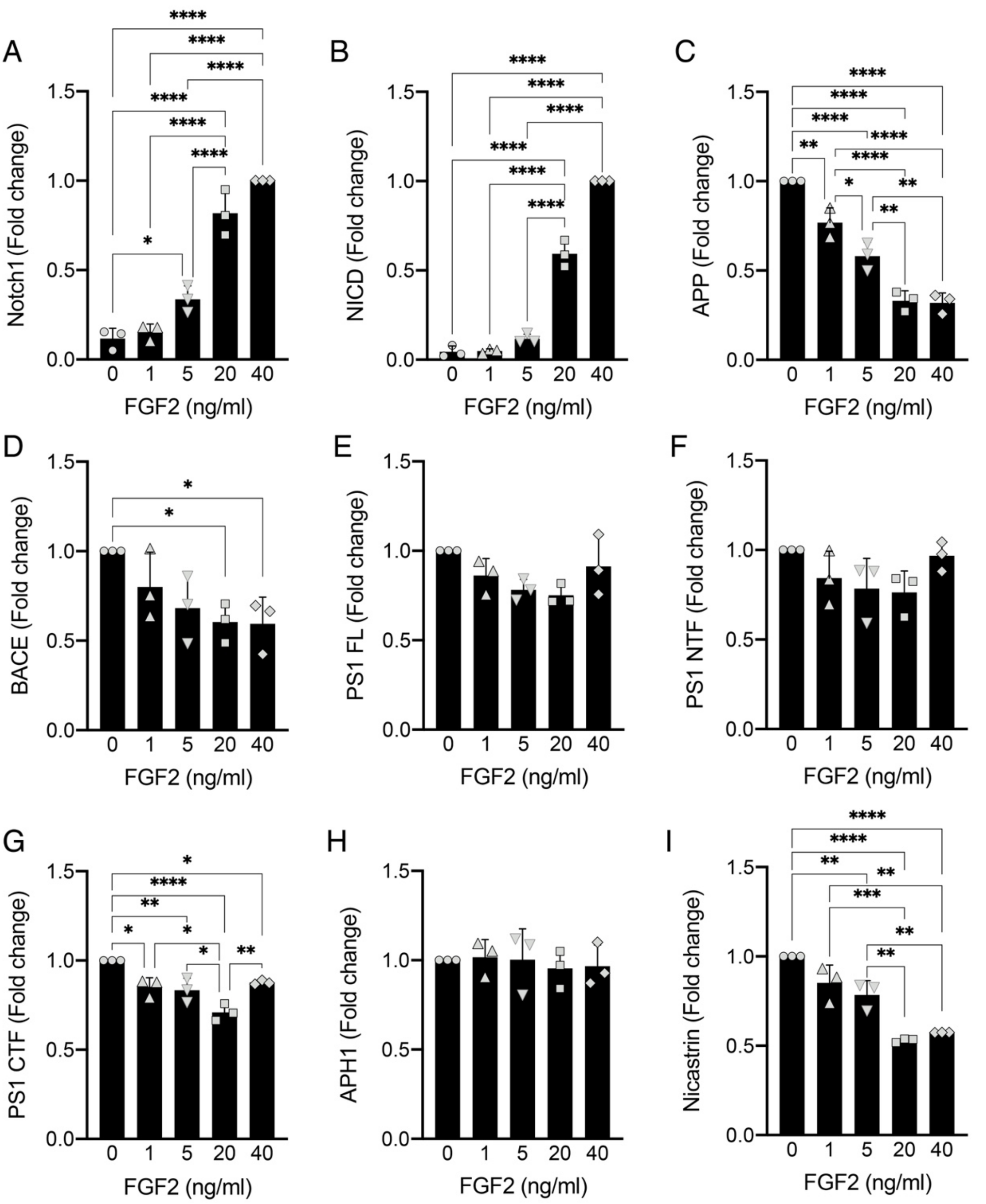

Supplementary Figure 5. Quantification of the protein levels in Figure 3a & b. (**A**) Notch1. (**B**) NICD. (**C**) APP. (**D**) BACE1. (**E**) PS1 FL. (**F**) PS1 NTF. (**G**) PS1 CTF. (**H**) APH-1. (**I**) Nicastrin. n = 3/group; **** $P < 0.0001$, *** $P < 0.001$, ** $P < 0.01$, *$P < 0.05$; one-way ANOVA, Tukey's multiple comparisons test. (A & B) The protein levels are calculated as those at 40 ng/ml FGF2 as 1. (C – I) The protein levels are calculated as those at 0 ng/ml FGF2 as 1.

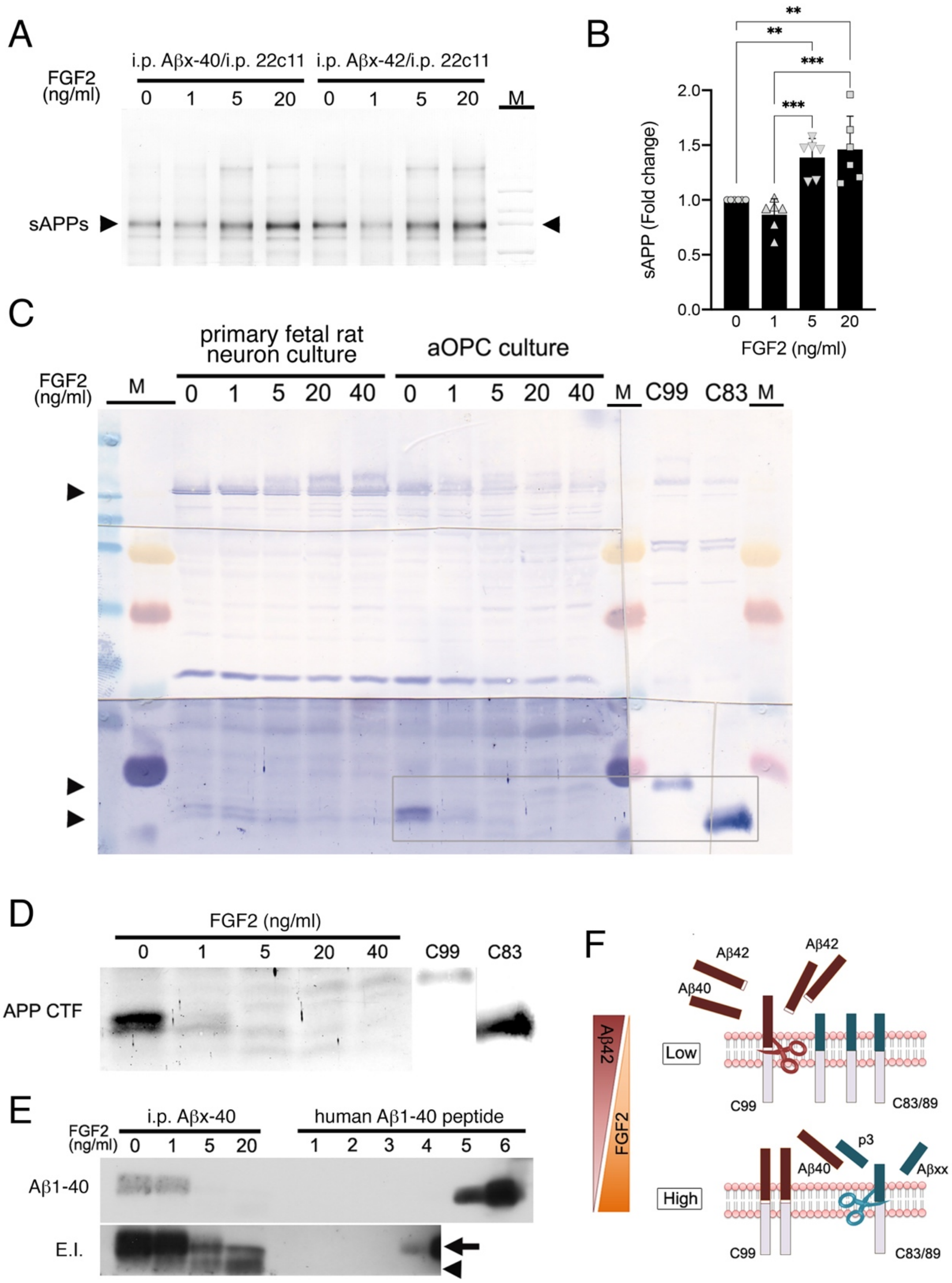

Supplementary Figure 6. Effects of FGF2 on APP processing in cultured aOPCs. (**A**) Analysis of secreted APP (sAPP) in the conditioned media of aOPCs. In parallel with Aβx-40 or Aβx-42 immunoprecipitation (i.p.) (**Figure 3c**), sAPP, which is generated from APP by α- or β-secretases, was immunoprecipitated from the

conditioned media containing 0, 1, 5, or 20 ng/ml FGF2, in which aOPCs were cultured for 24 h using an anti-APP (22c11) antibody. The resulting immunoprecipitate samples were analyzed by Western blot using the 22c11 antibody to quantify the levels of sAPP. The arrowheads indicate the predicted sAPP band positions (at around 85 kDa). M: molecular markers.

(**B**) Quantification of the levels of sAPP in the conditioned media (n = 6/group; $^{***}P$ <0.001, $^{**}P$ <0.01; one-way ANOVA, Tukey's multiple comparisons test. The protein levels are calculated as those at 0 ng/ml FGF2 as 1.).

(**C & D**) Effects of FGF2 on APP-CTF levels in cultured fetal rat neurons and aOPCs. **C**. A full scan image of a Western blot analyzing cell lysates of primary fetal rat hippocampal neurons (cultured for 3 weeks in vitro) and aOPCs cultured in medium containing 0, 1, 5, 20, or 40 ng/ml FGF2 for 5 days. Bands were detected using an anti-APP-C antibody. To detect the exact band positions of APP-Cs, CHO cell lysates overexpressing recombinant human APP-C83 (C83) or APP-C99 (C99) were also analyzed. Upper arrowhead: full-length APP. Full-length APP expression is regulated in an FGF2-dependent manner, but is differently regulated between cultured fetal rat neurons and aOPCs. Note that full-length APP levels in aOPCs cultured in medium containing 0 ng/ml FGF2 were similar to those in fetal neurons. The lower two arrowheads indicate the band positions of C99 and C83. Note that the regulation of APP-Cs was considerably different between fetal rat neurons and aOPCs. **D**. The area of the grey rectangle in panel C has been trimmed and enlarged.

(**E**) Levels of Aβ1-40 and p3-like peptides in the conditioned aOPC media. Aβ1-40 and p3-like peptides were immunoprecipitated using an anti-Aβ antibody (Aβx-40) from the conditioned media containing 0, 1, 5, or 20 ng/ml FGF2 in which aOPCs were cultured for 24 h. Upper: upon brief exposure of the Western blot membrane, only Aβ1-40 bands were detected. Lower: upon longer exposure (E.I.: enhanced image), additional bands (arrowhead), most likely representing p3-like peptides, became detectable just below the Aβ1-40 bands (arrow). Note that the levels of the p3-like peptide increased as FGF2 concentrations increased. Lanes 1–6 correspond to 12, 60, and 120 pM and 1.2, 12, and 120 nM human Aβ1-40 peptides, respectively.

(**F**) Hypothetical representation of the FGF2-dependent substrate preference of γ-secretase in cultured aOPCs. At 0 (or 1) ng/ml FGF2, γ -secretase (red scissors) in aOPCs prefers amyloidogenic APP-C99, resulting in increased secretion of Aβ1-40 and Aβ1-42. In striking contrast, at higher concentrations (20–40 ng/ml) of FGF2, γ -secretase (blue scissors) prefers non-amyloidogenic APP-C83/APP-C89, resulting in the relative accumulation of APP-C99.

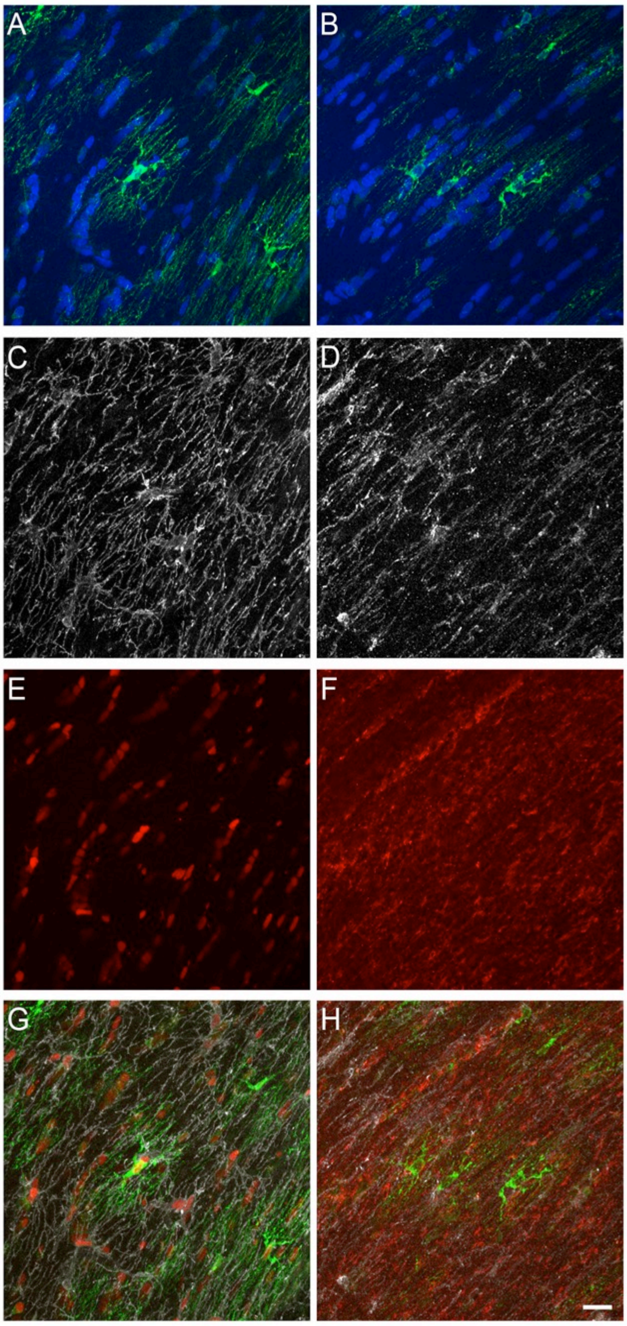

Supplementary Figure 7. Plexin-B3$^+$ aOPCs in the rat corpus callosum.

Brain sections from normal adult rats were immunostained using anti-plexin-B3 (**A** & **B**), anti-NG2 (**C** & **D**), anti-olig2 (**E**), or anti-MBP (**F**) antibodies. Merged images are shown in **G** & **H**. Nuclear staining (blue, Hoechst 33258) is also shown in **A** and **B**. In the corpus callosum, plexin-B3$^+$ aOPCs were negative for MBP and NG2, but positive for olig2. Scale bar: 20 μm.

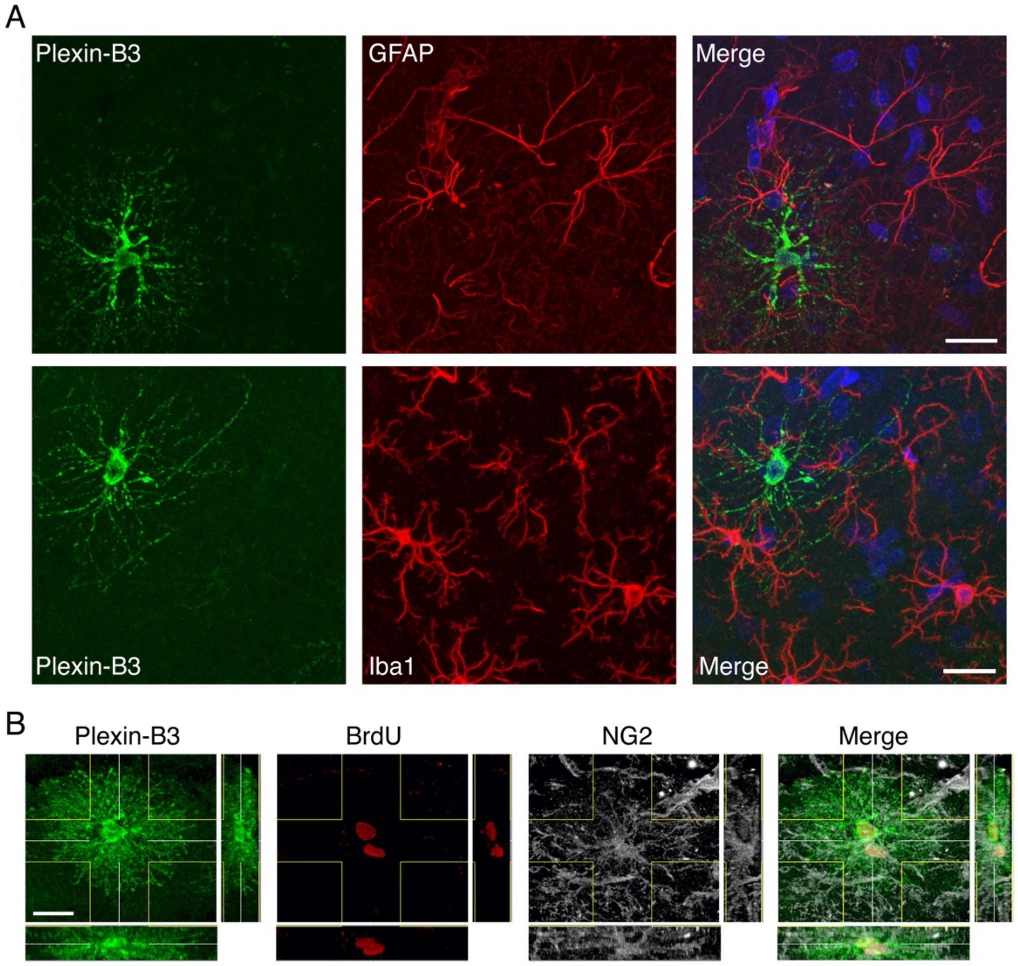

Supplementary Figure 8. Plexin-B3$^+$ aOPCs in the rat cortex.

(**A**) Plexin-B3$^+$ aOPCs were negative for GFAP and Iba1. Brain sections from normal adult rats were immunostained using anti-plexin-B3 and anti-GFAP or anti-Iba1 antibodies. Nuclear staining (blue, Hoechst 33258) is shown in the "Merge" panels. Scale bar: 20 μm.

(**B**) A pair of plexin-B3$^+$/BrdU$^+$ and NG2$^+$/BrdU$^+$ cells, most likely formed by asymmetrical aOPC division. Brain sections from normal adult rats treated with BrdU were immunostained using anti-plexin-B3, anti-BrdU, and anti-NG2 antibodies. The merged image is shown in the right-most panel. Scale bar: 20 μm.

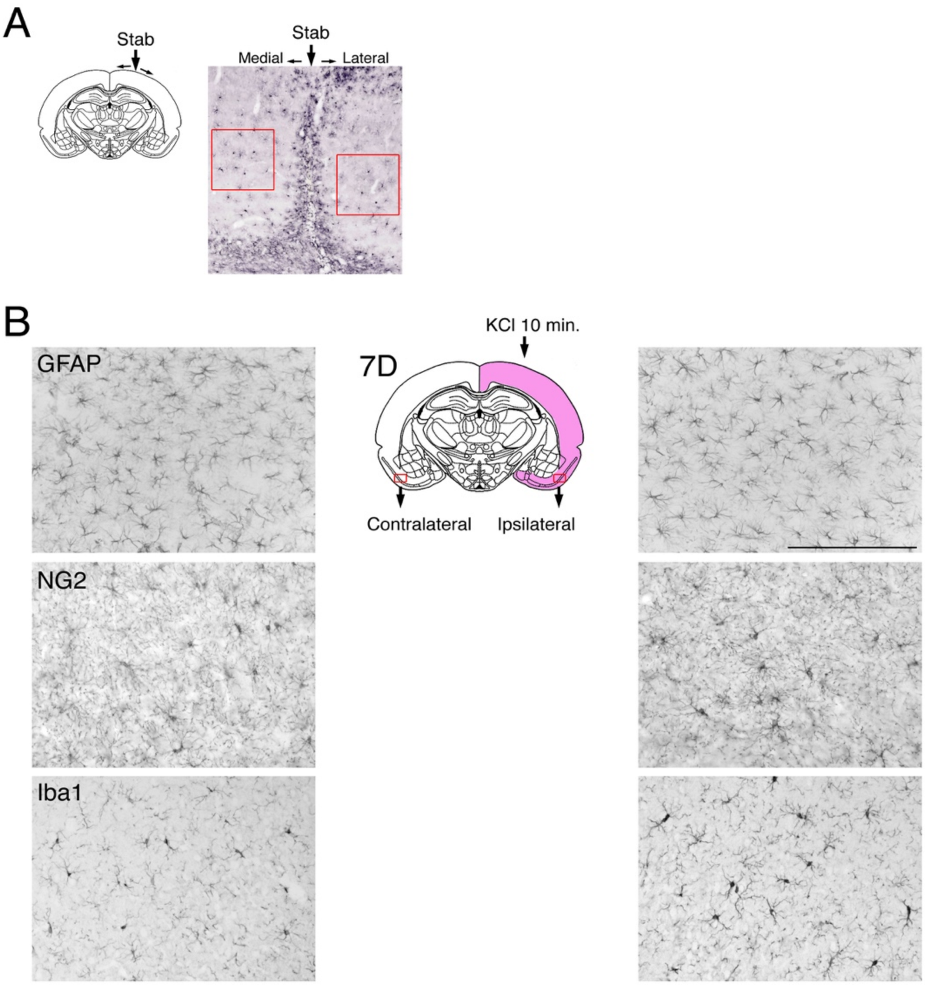

Supplementary Figure 9. Areas for quantitative cell counting in the brain injury models.

(**A**) For the stab wound model (**Figure 5a & b**), two 200 μm squares were defined in the cortex, approximately 30 ~ 50 μm apart medially and laterally from the stab lesion. The image of plexin-B3 staining is the same as shown in **Figure 5a** at 7 days.

(**B**) For the KCl injury model (**Figure 5e**), two 440 μm x 330 μm rectangles were defined in the ipsilateral and contralateral remote cortex, as shown in the schematic figure. Brain sections were immunostained using antibodies against plexin-B3, GFAP, NG2, or Iba1. Scale bar: 200 μm.

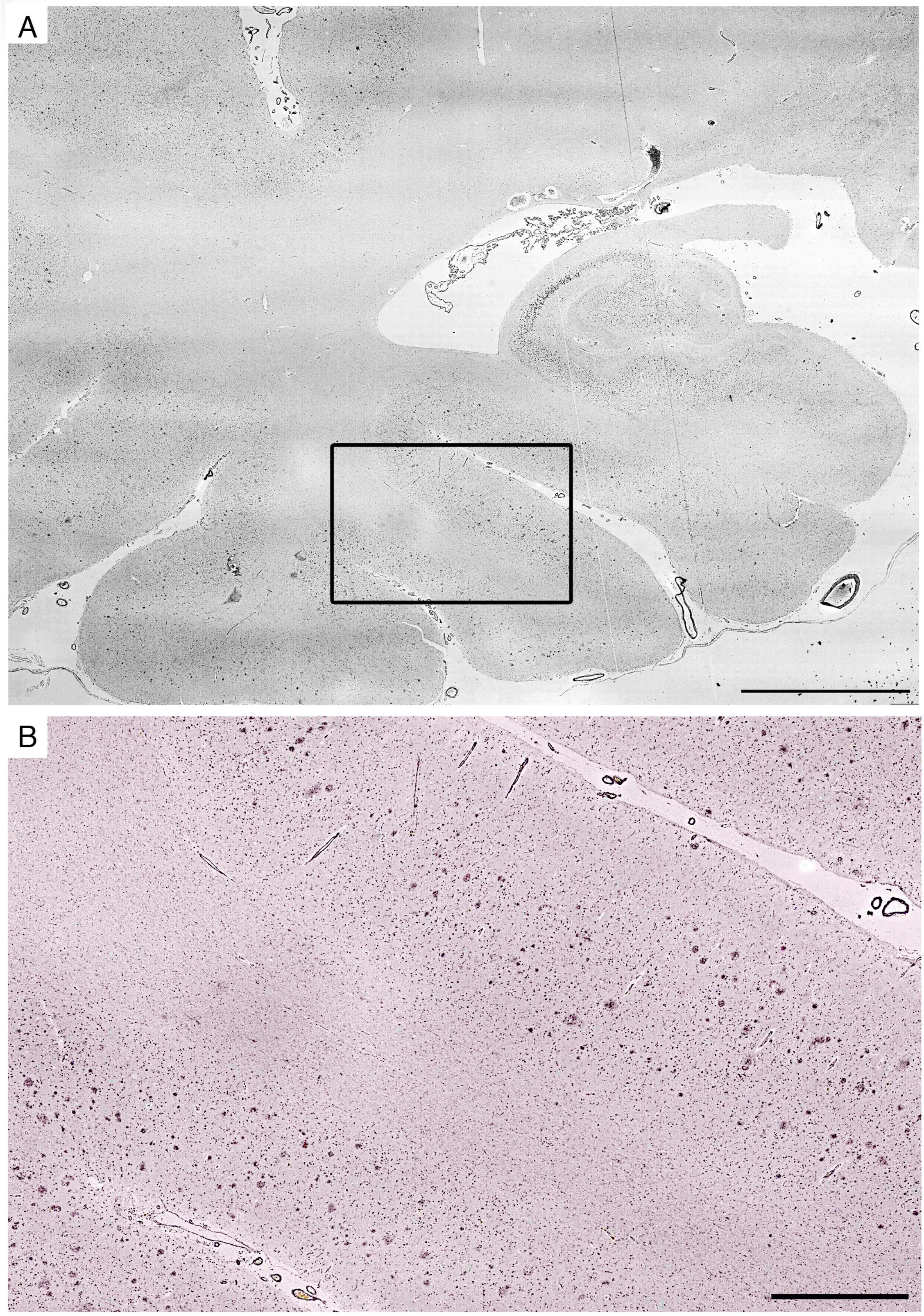

Supplementary Figure 10. Cortical distribution of plexin-B3^{+} senile plaques in the AD brain

Immunohistochemical analysis of an AD brain using an anti-plexin-B3 polyclonal antibody (R&D), which stained mainly cortical structures (**A** and **B**), except for dot-like structures distributed throughout the entire brain (**B**). Scale bar: **A**, 5 mm; **B**, 1 mm.

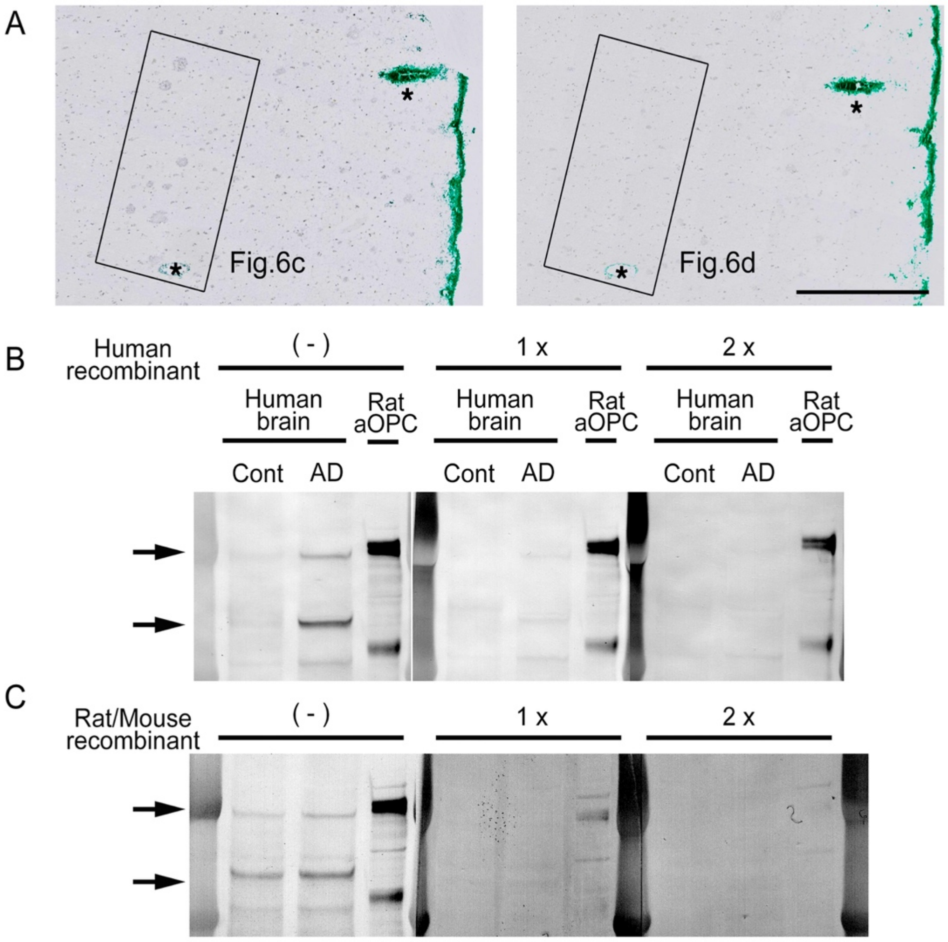

D

	Human	Rat
Human recombinant	✓	×
Rat/Mouse recombinant	✓	✓

Supplementary Figure 11. Specificity of the anti-plexin-B3 polyclonal antibody.
(**A**) Original images of **Figure 6c & d**. Serial sections from an AD brain were immunostained using an anti-plexin-B3 polyclonal antibody (R&D) without (left) or with (right) 5x recombinant human plexin-B3 (His45 – Gln1255 (Glu1156Asp) with a C-terminal 6-His tag) pretreatment (R&D) overnight. Note that pre-absorption successfully eliminated plaque staining, but not the dot-like staining, suggesting that the latter is most likely non-specific. Asterisks indicate blood vessels colored using a green marker before sectioning. Scale bar: 100 μm.
(**B**) Western blot analysis for the specificity of the anti-plexin-B3 polyclonal antibody (R&D) against human plexin-B3. The antibody was pretreated without (-) or with (1x or 2x) recombinant human plexin-B3 (His45 – Gln1255 (Glu1156Asp) with a C-terminal

6-His tag) (R&D) overnight and used for the Western blot analysis. Note that two major bands of human plexin-B3 in the Sarkosyl-soluble fractions, but not rat plexin-B3 expressed in the cultured aOPCs, were successfully eliminated by the pre-absorption.
(**C**) Western blot analysis for the specificity of the anti-plexin-B3 polyclonal antibody (R&D) against rat plexin-B3. The antibody was pretreated without (-) or with 1x or 2x recombinant rat/mouse plexin-B3 (His25-Gln1235 with a C-terminal 6-His tag) (R&D) overnight and used for the Western blot analysis. Note that both, human and rat plexin-B3 bands were successfully eliminated by the pre-absorption.
(**D**) Summary of the pre-absorption study results.

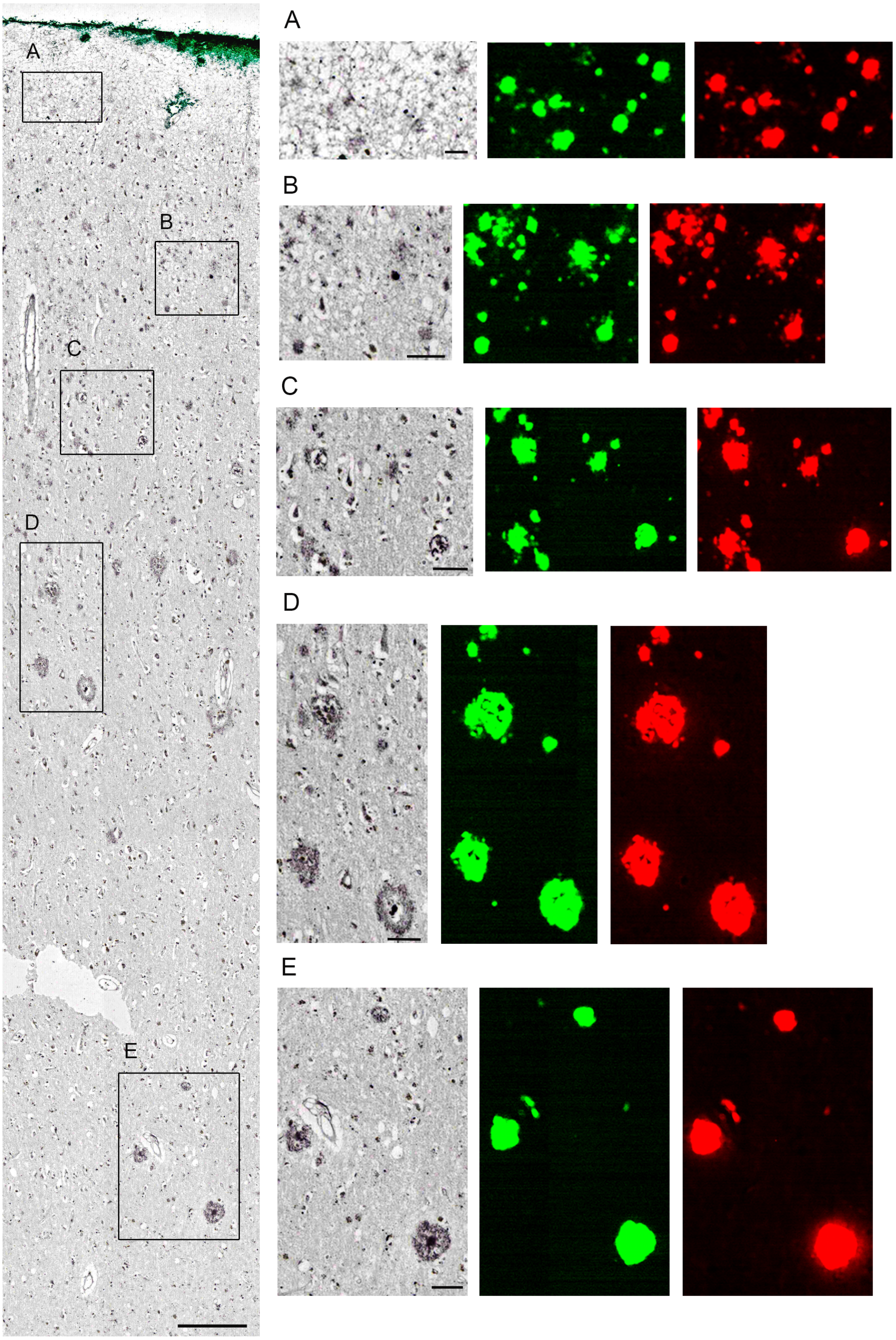
A
B
C
D
E
A
B
C
D
E

Supplementary Figure 12. Spatial relationship between plexin-B3^{+} and Aβ^{+} areas. Immunostaining of an AD brain using an anti-plexin-B3 polyclonal antibody (R&D) and antibodies against total Aβ (4G8, green) and Aβ1-42 (red). Images were digitalized using a virtual slide system (VS120; Olympus, Tokyo, Japan). Almost all the SPs were co-immunolabelled by the anti-plexin-B3 antibody. Scale bars: left-most panel, 200 μm; **A**, 20 μm; **B**, **C**, **D**, and **E**, 50 μm.

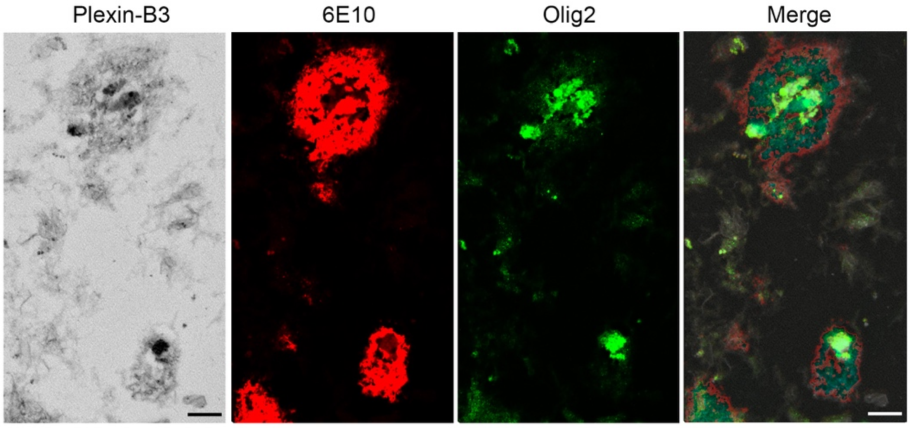

Supplementary Figure 13. Olig2 immunoreactivity of plexin-B3$^+$ senile plaques. Immunostaining of an AD brain using an anti-plexin-B3 polyclonal antibody (R&D) and antibodies against total Aβ (6E10, red) and olig2 (green). The images for plexin-B3 were digitalized using a virtual slide system (VS120; Olympus, Tokyo, Japan). Olig2$^+$ senile plaques were occasionally found in AD brains, and were all plexin-B3$^+$. Scale bar, 15 μm.

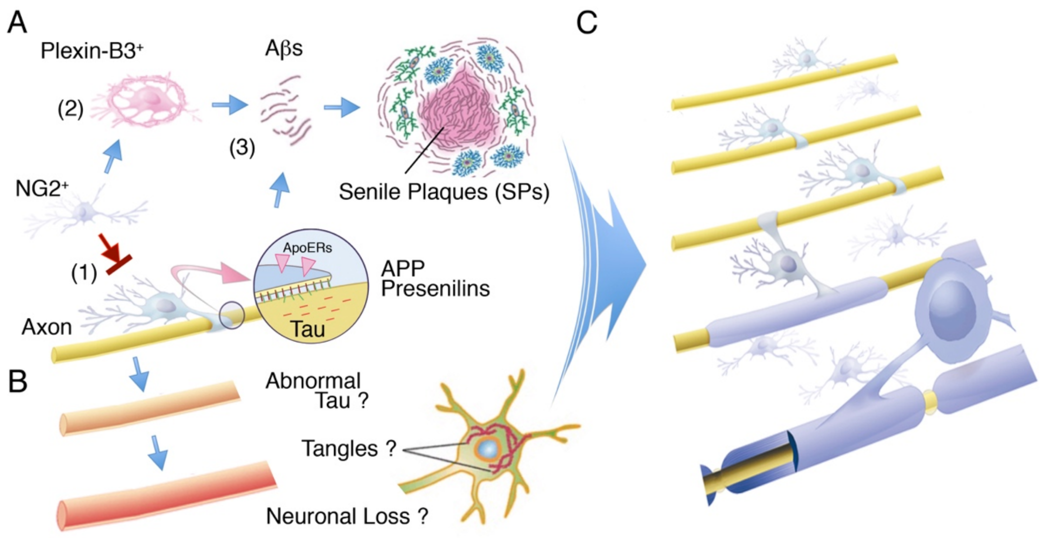

Supplementary Figure 14. Possible sporadic AD pathology: implications from the present study.

(**A**) A type of demyelination or dysmyelination occurs in the sporadic AD cortex (1), resulting in defective plexin-B3$^+$ aOPC differentiation (2) and extracellular Aβ accumulation (3). ApoERs: apolipoprotein E receptors expressed on a myelinating oligodendrocyte.

(**B**) AD-type demyelination or dysmyelination may eventually promote axonal dysfunction, neurological disabilities, neurofibrillary tangle formation in neurons, and neuronal loss.

(**C**) Fine control of cortical myelination in aged brains may be an essential requirement of effective AD therapy.

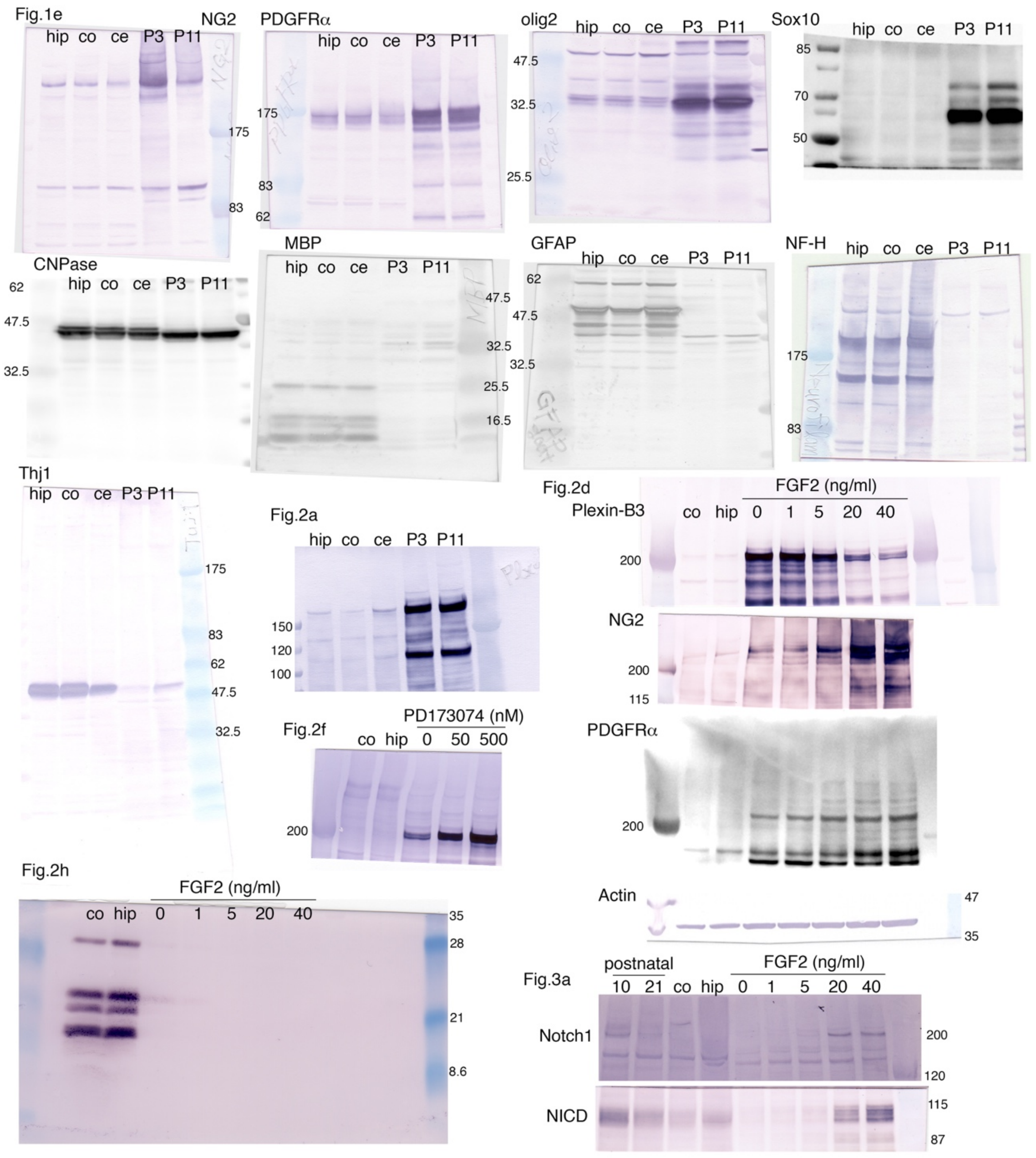

Supplementary Figure 15. Full images of Western blot results in Figure 1e, 2a, 2d, 2f, & 3a.

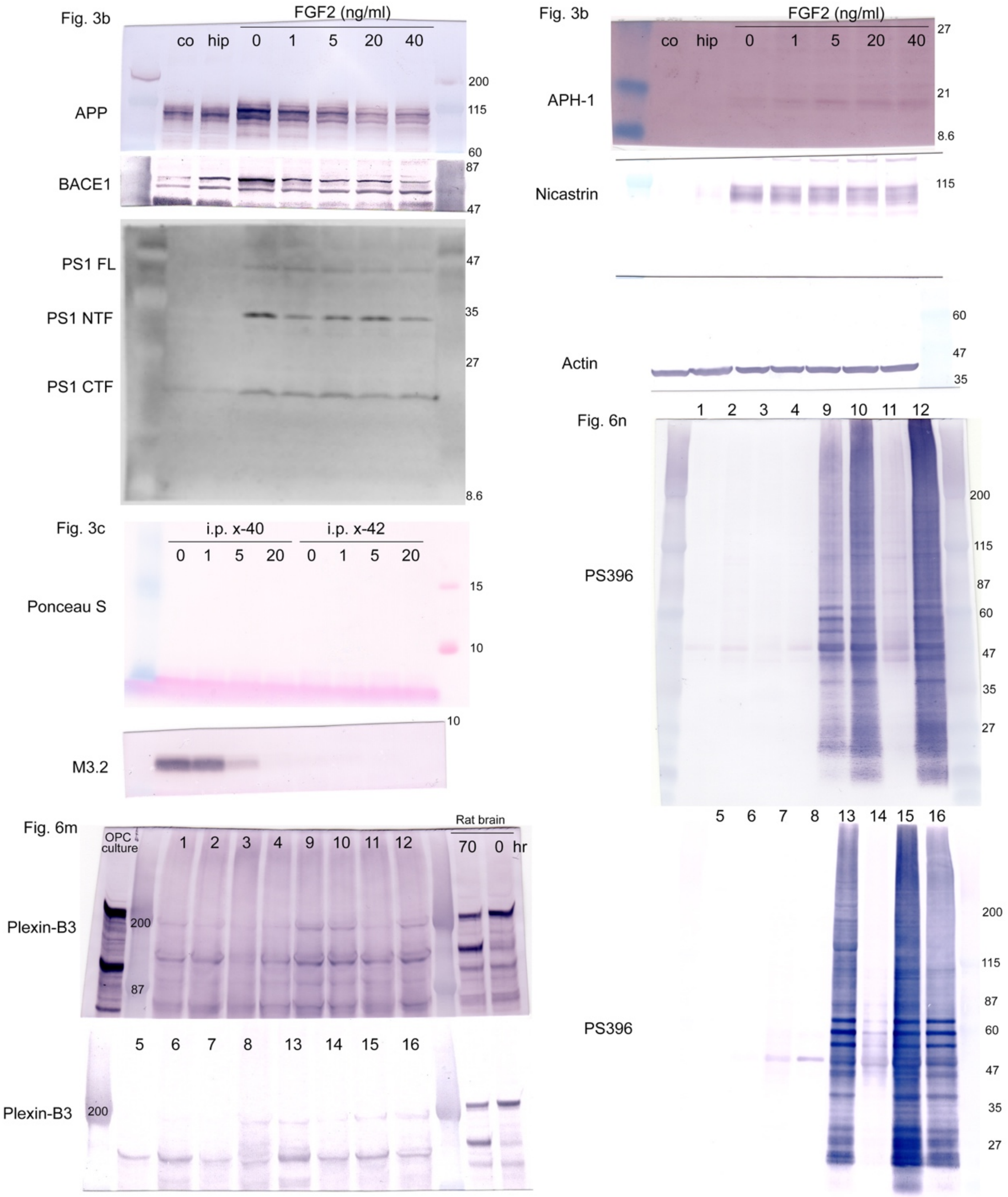

Supplementary Figure 15. Full images of Western blot results in Figure 3b, 3c, 6m, & 6n.

Brain area	Debris	Sup NG2%
Hippocampus	+	93.5 ± 4.3
Olfactory bulb	++	87.7 ± 9.0
Amygdala	++	92.2 ± 5.0
Striatum	++	83.4 ± 13.7
Cortex	+++	88.5 ± 6.5
Medulla oblongata	++++	78.4 ± 23.8
Cerebellum	++++	84.3 ± 3.4
Thalamus and midbrain	+++++	85.4 ± 13.8
Spinal Cord	+++++	91.7 ± 14.4

Supplementary Table 1. Isolation and culture of aOPCs from various regions of adult rat brains.

Using the Paxinos and Watson atlas (http://labs.gaidi.ca/rat-brain-atlas/) as a guide, different brain regions were dissected from adult SD rats according to the step-by-step protocol by Spijker[58]. aOPCs were cultured for 5 days, and the proportion of $NG2^+$ cells (Sup NG2%) was determined based on the ratio (n = 3/group) of immunolabeled cells to total (TO-PRO-3^+) cells. The amount of debris (in comparison with the attached [alive] cells on the culture dishes) was scored based on the impression of the observers at the time of primary culture.

FGF2	20 ng/ml	0 ng/ml
$NG2^+$	> 90 %	21.1± 1.94 %
Plexin-$B3^+$	1.07 ± 0.27%	29.5 ± 6.92 %
$olig2^+$	> 99 %	> 99 %

Supplementary Table 2. Effects of FGF2 (0 or 20 ng/ml) on the expression of aOPC markers.

After 5 days of culture in medium with 0 or 20 ng/ml FGF2, the cells were fixed and immunostained with antibodies against NG2, plexin-B3, and/or olig2, and the number of cells positive for each aOPC marker was counted (n = 5/group). The total cell numbers were counted using TO-PRO-3 or Hoechst 33258. The proportions of each aOPC marker-positive cells are shown. Note that $NG2^+$ cells were generally plexin-B3-negative and vice versa (see also **Figure 2b–d**), and that more than 99% of living cells were $olig2^+$ under both conditions (see also **Figure 2g**).

Antibody	Species	Source	Cat.#	Dilutions
NG2	Rabbit	Millipore	AB5320	1: 500(IHC), 1: 1,000(WB)
NG2	Mouse	Millipore	MAB5384	1: 200(IHC)
PDGFRα	Rabbit	Santa Cruz	sc-338	1: 500(IHC), 1: 1,000(WB)
Olig2	Rabbit	Millipore	AB9610	1: 500(IHC), 1: 1,000(WB)
O4	Mouse	Millipore	MAB345	1: 66(IHC)
GFAP	Mouse	Millipore	MAB3402	1: 2,000(IHC)
GFAP	Rabbit	DAKO	Z0334	1: 2,000(IHC), 1: 2,000(WB)
Tuj1	Mouse	Covance	MMS-435P	1: 1,000(WB)
Neurofilament H	Rabbit	Millipore	AB1989	1: 200(IHC), 1: 1,000(WB)
Actin	mouse	BioVision	3598-100	1: 1,000(WB)
Plexin B3	Sheep	R&D	AF6879	1: 200~1,000(IHC), 1: 2,000(WB)
Notch 1	Goat	Santa Cruz	sc-6015	1: 2,000(WB)
NICD	Rabbit	Cell Signaling	4147	1: 2,000(WB)
GAPDH	Mouse	Santa Cruz	sc-32233	1: 1,000(WB)
APP(22C11)	Mouse	Millipore	MAB10424	1: 2,000(WB)
APP(C1/6.1)	Mouse	BioLegend	802802	1: 1,000(WB)
BACE1	Rabbit	Calbiocam	195111	1: 2,000(WB)
Presenilin 1	Mouse	Millipore	MAB5232	1: 2,000(WB)
Human Aβ17-24 (4G8)	Mouse	Covance	SIG-39220	1: 1,000(WB)
Nicastrin	Rabbit	Signo Biological Inc	11183-RP02	1: 2,000(WB)
Aph1(N-20)	Goat	Santa Cruz	sc-30240	1: 200(WB)
Rodent Aβ10-15 (M3.2)	Mouse	Covance	SIG-39155	1: 200(IHC), 1: 2,000(WB)
Aβ1-40	Rabbit	IBL	18580	1: 200(IHC), 1: 2,000(WB)
Aβ1-42	Rabbit	IBL	18582	1: 200(IHC), 1: 1,000(WB)
Tau [pS396]	Rabbit	BIOSOURCE	44-752G	1: 1,000(WB)
BrdU	Rat	serotec	OBT0030CX	1: 500(IHC)
Iba1	Rabbit	Wako	019-19741	1: 1,000(IHC)
Iba1	Rabbit	Wako	061-20001	1: 1,000(WB)
Sox10	Rabbit	Millipore	AB5727	1: 2,000(WB)
CNPase	Mouse	Millipore	MAB326	1: 1,000(WB)
MBP	Mouse	Millipore	MAB384-1ML	1: 500(IHC), 1: 1,000(WB)
CD68	Mouse	DAKO	M0814	1: 500(IHC)
Human Aβ1-16 (6E10)	Mouse	Covance	SIG-39300	1: 300(IHC)
Olig2	Rabbit	IBL	18953	1: 100(IHC)

Supplementary Table 3. List of antibodies used in the present study.

A

Case #	Age	Gender	Diagnosis	PMI	NFT	Amyloid	Weight	Region
1	63	M		2h 41min	I		1515	Hip
2	75	F		2h 6min	II		1280	Hip
3	78	M		19h 56min	II		1270	Hip
4	68	M		16h 2min	II		1460	Hip
5	78	F		46h 56min	I		1200	Hip
6	80	M	AD + DLB	17h 11min	V	B	1210	Hip
7	93	M	AD	19h 47min	VI	C	1080	Hip
8	94	M	AD	81h 33min	V	B	1290	Hip
9	96	F	AD	21h 43min	V	C	1140	Hip
10	88	F	AD	76h 46min	V	C	1190	Hip

B

Case #	Age	Gender	Diagnosis	PMI	NFT	Region
1	56	M		8h	I/I	BA6
2	60	M		11h 32min	I/I	BA6
3	87	M		2h 32min	II/III	BA6
4	57	M		9h 20min	I/I	BA6
5	79	M		3h 21min	III	BA6
6	81	M		4h 29min	III	BA6
7	58	M		16h 47min	II	BA6
8	77	M		1h 38min	III	BA6
9	87	M	AD	5h 50min	V/V	BA6
10	66	M	AD + DLB	16h	V/V	BA6
11	86	F	AD	7h 59min	IV/IV	BA6
12	59	F	AD + DLB	3h	VI/VI	BA6
13	92	M	AD	5h 8min	V	BA6
14	80	M	AD	13h 50min	IV	BA6
15	89	F	AD	4h 58min	VI	BA6
16	89	F	AD	4h 0min	IV	BA6

Supplementary Table 4. Demographic data of human brains.

(**A**) Demographic data corresponding to the samples used for immunohistochemical analysis. Ten brains from subjects (5 AD patients and 5 disease controls) autopsied at the Nitobe Memorial Nakano General Hospital were analyzed.

(**B**) Demographic data corresponding to samples used for the Western blot analysis. Sixteen frozen cortices from subjects (8 AD patients and 8 controls) autopsied at the Aichi Medical University were analyzed.

Age: age at death (years), M: male, F: female, Diagnosis: neuropathological diagnosis, DLB: diffuse Lewy body disease, PMI: postmortem interval, NFT: Braak neurofibrillary tangle stage, Amyloid: Braak amyloid stage, Weight: brain weight at autopsy (g), Hip: hippocampus, BA6: Brodmann area 6.
