# Supplementary material for: Essential roles of plexin-B3+ oligodendrocyte precursor cells in the pathogenesis of Alzheimer’s disease
Source: Commun Biol. 2021 Jul 15;4:870. doi: 10.1038/s42003-021-02404-7 (PMC8282672; doi:10.1038/s42003-021-02404-7)
Supplement: Supplementary file 3 — Description of Additional Supplementary Files [file 42003_2021_2404_MOESM3_ESM.pdf]

Description of Additional Supplementary Files

Title: Supplementary Data 1
Description: Correspondence table for Figure 1F,

Title: Supplementary Data 2
Description: Correspondence table for Supplementary Figure 2a

Title: Supplementary Data 3
Description: Correspondence table for Supplementary Figure 2b

Title: Supplementary Data 4
Description: Correspondence table for Supplementary Figure 4c

Title: Supplementary Data 5
Description: Correspondence table for Supplementary Figure 3a & Supplementary Figure 4d

Title: Supplementary Data 6
Description: Correspondence table for Supplementary Figure 3b & Supplementary Figure 4e
