# Supplementary material for: Essential roles of plexin-B3+ oligodendrocyte precursor cells in the pathogenesis of Alzheimer’s disease
Source: Commun Biol. 2021 Jul 15;4:870. doi: 10.1038/s42003-021-02404-7 (PMC8282672; doi:10.1038/s42003-021-02404-7)
Supplement: Supplementary file 10 — Reporting Summary [file 42003_2021_2404_MOESM10_ESM.pdf]

nature research

Corresponding author(s): Yoshitaka Tatebayashi

Last updated by author(s): May 20, 2021

Reporting Summary

Nature Research wishes to improve the reproducibility of the work that we publish. This form provides structure for consistency and transparency in reporting. For further information on Nature Research policies, see our Editorial Policies and the Editorial Policy Checklist.

Statistics

For all statistical analyses, confirm that the following items are present in the figure legend, table legend, main text, or Methods section.

n/a	Confirmed	
☐	☒	The exact sample size (*n*) for each experimental group/condition, given as a discrete number and unit of measurement
☐	☒	A statement on whether measurements were taken from distinct samples or whether the same sample was measured repeatedly
☐	☒	The statistical test(s) used AND whether they are one- or two-sided<br>*Only common tests should be described solely by name; describe more complex techniques in the Methods section.*
☒	☐	A description of all covariates tested
☒	☐	A description of any assumptions or corrections, such as tests of normality and adjustment for multiple comparisons
☒	☐	A full description of the statistical parameters including central tendency (e.g. means) or other basic estimates (e.g. regression coefficient) AND variation (e.g. standard deviation) or associated estimates of uncertainty (e.g. confidence intervals)
☒	☐	For null hypothesis testing, the test statistic (e.g. *F*, *t*, *r*) with confidence intervals, effect sizes, degrees of freedom and *P* value noted<br>*Give P values as exact values whenever suitable.*
☒	☐	For Bayesian analysis, information on the choice of priors and Markov chain Monte Carlo settings
☒	☐	For hierarchical and complex designs, identification of the appropriate level for tests and full reporting of outcomes
☒	☐	Estimates of effect sizes (e.g. Cohen's *d*, Pearson's *r*), indicating how they were calculated

Our web collection on statistics for biologists contains articles on many of the points above.

Software and code

Policy information about availability of computer code

Data collection	LAS-3000 Mini imager (GE Healthcare), Multi Gauge V2.3 software (GE Healthcare), Agilent Microarray Scanner (Agilent Technologies), HiSeq 2500 platform (Illumina), confocal microscopy (Zeiss), virtual slide system (VS120; Olympus)
Data analysis	GraphPad PRIZM 8 (GraphPad Software), ImageJ, Zen2011 imaging software (Zeiss), Agilent Feature Extraction software 9.5.3.1 (Agilent Technologies), TopHat tool (version 2.0.14), Spike2 software (Cambridge Electronic Device)

For manuscripts utilizing custom algorithms or software that are central to the research but not yet described in published literature, software must be made available to editors and reviewers. We strongly encourage code deposition in a community repository (e.g. GitHub). See the Nature Research guidelines for submitting code & software for further information.

Data

Policy information about availability of data

All manuscripts must include a data availability statement. This statement should provide the following information, where applicable:

- Accession codes, unique identifiers, or web links for publicly available datasets
- A list of figures that have associated raw data
- A description of any restrictions on data availability

We state that all figures have associated raw data and there are no restrictions on data availability.

Field-specific reporting

Please select the one below that is the best fit for your research. If you are not sure, read the appropriate sections before making your selection.

[x] Life sciences [ ] Behavioural & social sciences [ ] Ecological, evolutionary & environmental sciences

For a reference copy of the document with all sections, see nature.com/documents/nr-reporting-summary-flat.pdf

Life sciences study design

All studies must disclose on these points even when the disclosure is negative.

Sample size	No statistical method was used to predominate sample size.
Data exclusions	No data was excluded from the analyses.
Replication	All attempts at replication were successful.
Randomization	The samples were not randomized for the experiments.
Blinding	Blinding was performed in Figure 5e & 5f. In the other experiments, no blinding was performed.

Reporting for specific materials, systems and methods

We require information from authors about some types of materials, experimental systems and methods used in many studies. Here, indicate whether each material, system or method listed is relevant to your study. If you are not sure if a list item applies to your research, read the appropriate section before selecting a response.

Materials & experimental systems

n/a	Involved in the study	
[ ]	[x]	Antibodies
[x]	[ ]	Eukaryotic cell lines
[x]	[ ]	Palaeontology and archaeology
[ ]	[x]	Animals and other organisms
[ ]	[x]	Human research participants
[x]	[ ]	Clinical data
[x]	[ ]	Dual use research of concern

Methods

n/a	Involved in the study	
[x]	[ ]	ChIP-seq
[x]	[ ]	Flow cytometry
[x]	[ ]	MRI-based neuroimaging

Antibodies

Antibodies used	All antibodies used were described in Supplementary Table 3.
Validation	Original validation of anti-plexin-B3 polyclonal antibody was performed in Supplementary Figure 11. The other antibodies were validated as noted on manufacturer's website, and most of the antibody was confirmed in the literature.

Animals and other organisms

Policy information about studies involving animals; ARRIVE guidelines recommended for reporting animal research

Laboratory animals	Female Sprague-Dawley (SD) rats (> 4 months old) were used in our studies except for the Western Blotting study shown in Figure 3a.
Wild animals	N/A
Field-collected samples	N/A
Ethics oversight	All protocols were approved by the Tokyo Metropolitan Institute of Medical Science Animal Care and Use Committee.

Note that full information on the approval of the study protocol must also be provided in the manuscript.

Human research participants

Policy information about studies involving human research participants

Population characteristics	Post-mortem brain tissue from AD patients and non-neurological controls were collected.

Recruitment	Permission of autopsy and of subsequent use for research was obtained from the next to kin of the patients in Nitobe Memorial Nakano General Hospital and Aichi Medical University.
Ethics oversight	All tissues were collected in accordance with Institutional Review Board-approved guidelines. The study was approved by ethics committee of Tokyo Metropolitan Institute of Medical Science.

Note that full information on the approval of the study protocol must also be provided in the manuscript.
